# Randomised controlled trial of a very brief nurse-delivered intervention followed by a digital intervention to support medication adherence and reduce blood pressure in people prescribed treatment for hypertension in primary care: protocol for the Programme on Adherence to Medication (PAM) trial

**DOI:** 10.3310/nihropenres.14136.1

**Published:** 2026-02-25

**Authors:** Stephen Sutton, James Jamison, Katja Beardsell, Micah Fineberg, Raquel Conceição, Venus Mirzaei, Catalina Trama Alvarez, Sonia Shpendi, James Brimicombe, Debi Bhattacharya, Wendy Hardeman, Felix Naughton, Miranda Van Emmenis, Helen Eborall, Anna De Simoni, Amrit Takhar, Pankaj Gupta, Prashanth Patel, Cecilia Mascolo, Andrew Toby Prevost, Joana Carvalho de Vasconcelos, Jonathan Mant, Simon Griffin, Richard J McManus, Stephen Morris

**Affiliations:** 1 Primary Care Unit, Department of Public Health and Primary Care, University of Cambridge, Cambridge, CB2 0SR, UK; 2 Division of Psychiatry, University College London, London, W1T 7NF, UK; 3 Department of Psychology, Counselling and Therapy, School of Psychology and Public Health, La Trobe University, Melbourne, Victoria, Australia; 4 Health Practice, Genesis Analytics, Johannesburg, 2196, South Africa; 5 Cambridgeshire and Peterborough NHS Foundation Trust, Cambridge, CB4 1PX, UK; 6 Specialist Children and Young People's Services, East London NHS Foundation Trust, London, E15 4PT, UK; 7 Frontiers Media SA, Lausanne, 1005, Switzerland; 8 School of Psychology, University of Sheffield, Sheffield, S1 4DP, UK; 9 School of Health Sciences, University of East Anglia, Norwich, NR4 7UL, UK; 10 Usher Institute, School of Population Health Sciences, University of Edinburgh, Edinburgh, EH16 4UX, UK; 11 Wolfson Institute of Population Health, Queen Mary University of London, London, E1 2AB, UK; 12 Wansford and Kings Cliffe Practice, Wansford, PE8 6PL, UK; 13 Department of Cardiovascular Sciences, University of Leicester, Leicester, LE1 7RH, UK; 14 Department of Computer Science and Technology, University of Cambridge, Cambridge, CB3 0FD, UK; 15 Clinical Research Facility, Moorfields Eye Hospital NHS Foundation Trust, London, EC1V 2PD, UK; 16 Brighton and Sussex Medical School, University of Brighton and University of Sussex, Brighton, BN1 9PX, UK

**Keywords:** Medication adherence, Hypertension, Blood pressure, Very brief intervention, Digital intervention, Primary care, Randomised controlled trial, Protocol

## Abstract

**Introduction:**

Hypertension is a global health challenge accounting for 8.5 million deaths worldwide despite the availability of low-cost pharmaceutical treatment. About 14.9% of people (9.9 million) registered with primary care practices in England and Wales are prescribed medication for hypertension. However, many patients with hypertension and associated conditions do not take their medications as prescribed. Non-adherence to antihypertensive medication is associated with increased risk of suboptimal blood pressure (BP) control, complications and all-cause mortality, and increased healthcare costs. The Programme on Adherence to Medication (PAM) trial will estimate the effectiveness and cost-effectiveness of a medication adherence intervention in patients prescribed medication for hypertension with poorly controlled blood pressure in primary care.

**Methods:**

A two-arm multicentre individually randomised controlled parallel group superiority trial recruiting patients prescribed medication for hypertension with poorly controlled BP in primary care practices in England and Wales. The target sample size is 542. Participants in the Intervention group will receive a very brief intervention delivered remotely (by telephone or video call) by a practice nurse or healthcare assistant followed by a digital intervention (text messaging or smartphone app) in addition to usual care; control group participants will receive usual care alone. The primary outcome is systolic BP measured at 12 months. Medication adherence will be measured by chemical adherence testing of urine samples and self-report. An economic evaluation and a process evaluation will be undertaken.

**Ethics and dissemination:**

The Cambridge East Independent Research Ethics Committee (REC reference 19/EE/0354), the Health Research Authority (HRA) and Health and Care Research Wales (HCRW) approved the trial. The findings will be disseminated to the scientific community, participating practitioners and patients, relevant patient groups and the public using a range of methods, including journal articles, conference presentations, newsletters and the Programme website.

**Trial registration number:**

The UK’s Clinical Trial Registry ISRCTN82013652

## Plain Language summary

About 15% of people (10 million) registered with primary care practices in England and Wales are prescribed treatment for hypertension (high blood pressure). However, many do not take their medications as prescribed, which may harm their health and increases health service costs. To address this problem, we need low-cost interventions that can reach all the people who need them.

Based on findings from our previous studies using a range of different research methods, we have developed a new intervention (the PAM intervention – Programme on Adherence to Medication) to support people with poorly controlled blood pressure to take their antihypertensive medication as prescribed. The intervention has two parts: a very brief intervention delivered by a practice nurse or healthcare assistant by telephone or video call, followed by a digital intervention (individually-tailored text messages for up to 14 months or a smartphone app).

We have already shown that the PAM intervention is feasible and acceptable. The next stage is to assess how well it works and how much it costs compared with usual care in a randomised controlled trial. In this trial, blood pressure and medication adherence will be measured in a large sample of participants recruited from primary care practices in England and Wales. This protocol describes the methods that will be used in the trial.

If the intervention is shown to increase medication adherence and reduce blood pressure and is found to be good value for money, it could be introduced in primary care practices throughout the UK.

## Introduction

Hypertension is a global health challenge accounting for 8.5 million deaths worldwide despite the availability of low-cost pharmaceutical treatment. ^1, 2^ About 14.9% of people (9.9 million) registered with primary care practices in England and Wales are prescribed medication for hypertension. ^3, 4^

However, many people with hypertension and associated conditions do not take their medications as prescribed. A meta-analysis involving 27 million patients found that the global prevalence of non-adherence to antihypertensive medication ranges from 27% to 40%. ^5^ Little information is available for the UK, though one study that used urine analysis reported a non-adherence rate of 41.6%. ^6^

Non-adherence to medication has been called a ‘worldwide problem of striking magnitude’ ^7^ and ‘one of the two largest unsolved gaps in health care’ ^8^ (the other being suboptimal prescribing of medication). Non-adherence to antihypertensive medication is associated with increased risk of suboptimal blood pressure (BP) control, complications and all-cause mortality, and increased healthcare costs. ^5, 9^

Primary care practitioners have an important role in supporting patients to adhere to their prescribed medication. ^10^ However, they lack time to provide ongoing support for adherence, and their time is expensive. A potential solution is for a practitioner such as a practice nurse to deliver a very brief intervention during a consultation and to use a digital intervention such as text messaging, smartphone app or website intervention to support subsequent adherence.

According to a recent report, 96% of UK adults use a mobile phone and in 93% of cases this is a smartphone ^11^; the corresponding figures for those aged 65 and above are 88% and 77%. This suggests that digital interventions have the potential to reach the majority of this population. Digital interventions have several advantages over traditional interventions: they can be fully automated; provide information that is highly tailored to the individual; be interactive; be available at any time; deliver support in real time; deliver support with high fidelity; and be easily updated.

Recent meta-analyses have reported promising findings for the effectiveness of nurse-led and digital interventions to improve medication adherence and reduce BP in people with hypertension. ^12– 20^ The interventions examined in these reviews varied widely in content and delivery, and the digital interventions that have been evaluated to date have not made full use of individual tailoring, interactivity and other features that may increase user engagement and potential effectiveness. ^21^ No reviews have focused on very brief interventions delivered by nurses or other practitioners in primary care.

We have developed an intervention comprising a very brief intervention delivered remotely by a practice nurse or healthcare assistant in primary care followed by a digital intervention (text messaging programme or smartphone app): the PAM (Programme on Adherence to Medication) intervention. The intervention was shown to be acceptable, feasible and potentially effective in supporting medication adherence and reductions in BP in patients prescribed medication for hypertension, compared with usual care alone. ^22^

The target group for the intervention is patients in primary care who do not take their antihypertensive medication as prescribed and have raised BP. There is substantial evidence that adherence is associated with BP control. ^23– 25^ In a study that used urine analysis to screen for 40 of the most commonly prescribed antihypertensive medications, there was an 11-point difference in mean systolic BP measured in the clinic between adherent and non-adherent patients. ^26^

The aim of this trial is to estimate the effectiveness and cost-effectiveness of the PAM intervention to improve medication adherence and reduce BP compared with usual care alone, to inform a decision on whether to implement the intervention in primary care.

## Methods

### Patient and public involvement

Patient and Public Involvement (PPI) was extensive before and during the PAM Programme. Prior to the start of the Programme, we organised two workshops to gather views from patients prescribed BP lowering medication. We also engaged with two local representatives and INsPIRE (the local PPI group). Four patients reviewed a summary of our application. We included a named PPI representative as a collaborator in the grant application and designated one of the Co-investigators as PPI lead.

During the trial, we will work with Cambridge University Hospitals (CUH) PPI panel and PPI representatives recruited for the Programme. Specific activities include reviewing documents (e.g. information sheets), reviewing the online training programme for practitioners, advising on development of the interventions and pre-testing the interventions.

The Programme Steering Committee includes an independent PPI member who contributed in various ways including testing and providing feedback on the app intervention.

Members of the public contributed to the research through events organised at the Cambridge Science Festival 2019 and the Cambridge Festival 2022.

When the results of the trial are available, we will consult our PPI panel for advice on how best to disseminate the findings, and we will encourage them to be directly involved in dissemination activities themselves.

### Trial design

**Figure 1.**
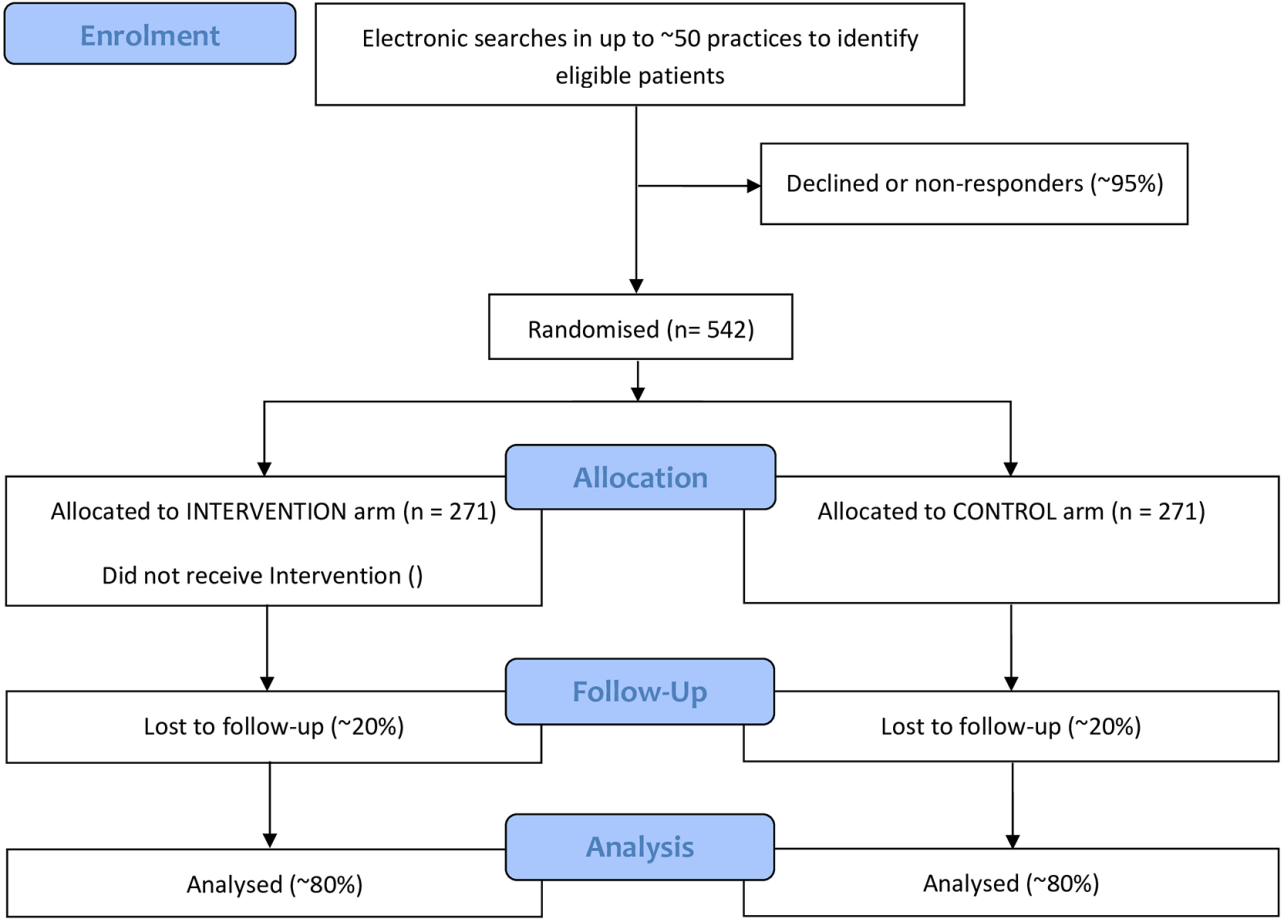
Trial flow chart.

A two-arm multicentre individually randomised controlled parallel group superiority trial of the PAM intervention versus usual care alone with the primary outcome of systolic BP measured at 12 months. (See Figure 1 for the trial flow chart.)

### Participants and recruitment

We will recruit primary care practices in England and Wales. Practices will be eligible to participate if they have at least 250 eligible patients with hypertension. We aim to recruit practices with a range of deprivation levels (defined by the Index of Multiple Deprivation derived from the practice postcode ^27^).

Practices will be required to have at least one eligible healthcare practitioner (i.e. nurse or healthcare assistant) who advises patients with hypertension during annual reviews, BP checks, medication reviews or similar consultations. To maximise generalisability, we have not specified additional practice-level eligibility criteria.

Participants will be eligible for inclusion if they: (a) have a diagnosis of hypertension; (b) have been prescribed at least one BP lowering or antihypertensive medication (e.g. ACE inhibitors, beta blockers, calcium channel blockers, diuretics, alpha1 blockers, alpha2 agonists) for at least one month before recruitment to the study, as confirmed by practice records; (c) have poorly controlled BP as indicated by at least two sequential clinical measures (i.e. clinic readings of BP >140/90 mmHg if under 80 years old and >150/90 mmHg if over 80 years old or home BP >135/85 mmHg if under 80 years and >145/85mmHg if over 80 years) obtained during the preceding 12 months or gaps in collecting repeat prescriptions (i.e. Cumulative Medication Gap, CMG > 0.20 for 12 months before recruitment); (d) have a good understanding of English; (e) own and are able to use a mobile phone; and (f ) have the capacity to provide informed consent.

Patients will be excluded if: (a) they have BP > 200/100mmHg or postural hypotension (>20mmHg systolic drop on standing); (b) they have a diagnosis of dementia or other cognitive difficulties that could affect study participation; (c) they have had a recent severe life-threatening event or are under treatment for a long-term health condition apart from hypertension, high cholesterol or type 2 diabetes (e.g. cancer); (d) they are taking part in another medication adherence intervention or digital intervention for behaviour change; (e) their BP is not managed by their GP practice; (f ) their GP, practice nurse or healthcare assistant feels that it is not appropriate to include them.

Eligible patients will be identified through practice database searches, conducted by a member of the practice staff, and checked for eligibility by a practice GP. Once identified, practices will invite patients into the trial opportunistically during usual care appointments or via text messages or postal invitations. Patients will be provided with printed information about the trial including a participant information sheet and consent form and given at least 24 hours to decide whether or not to participate. Those expressing an interest in participating by returning a reply slip will be telephoned by a member of the research team to address any questions or concerns. A remote baseline consultation will then be arranged between the healthcare practitioner and the participant and conducted by telephone or video call.

### Randomisation and blinding

During the baseline consultation, the practitioner will use an online system to check eligibility, obtain informed consent, obtain baseline questionnaire data, randomly allocate patients to the intervention or the control group, and, if the participant has been allocated to the intervention group, to deliver the very brief intervention (VBI). Eligible patients will be allocated randomly in a 1:1 ratio to receive the VBI followed by the digital intervention plus usual care or usual care only. The method of permuted blocks will be used with randomly varied block sizes of 4 and 6 and stratification by practitioner. Although the allocation sequence will be concealed, once a participant is allocated neither the practitioner nor the participant will be blind to group allocation. However, for some of the data collection and analysis procedures, it will be possible to maintain blinding. For example, staff at the laboratory who analyse the urine samples will not be aware of participants’ group allocation. Similarly, BP measures submitted by participants to the research team will be coded by staff who are not aware of the participants’ group allocation.

### Consent

As described above, informed consent will be obtained verbally by the practitioner during the baseline consultation. Verbal consent was chosen because neither the practitioner nor members of the research team will have face-to-face contact with the participant during the trial. However, in addition, we will attempt to obtain written informed consent by posting a consent form to each participant and asking them to sign and return it. All participants will be 18 years of age or older.

### Practitioner training

Practitioners will complete an online training session followed by an online or face-to-face meeting with a member of the research team. The training will focus on conducting the research procedures (e.g. taking informed consent) and delivering the VBI. Practitioners will be asked to repeat the online training before they start the baseline consultations.

### Intervention

**Figure 2.**
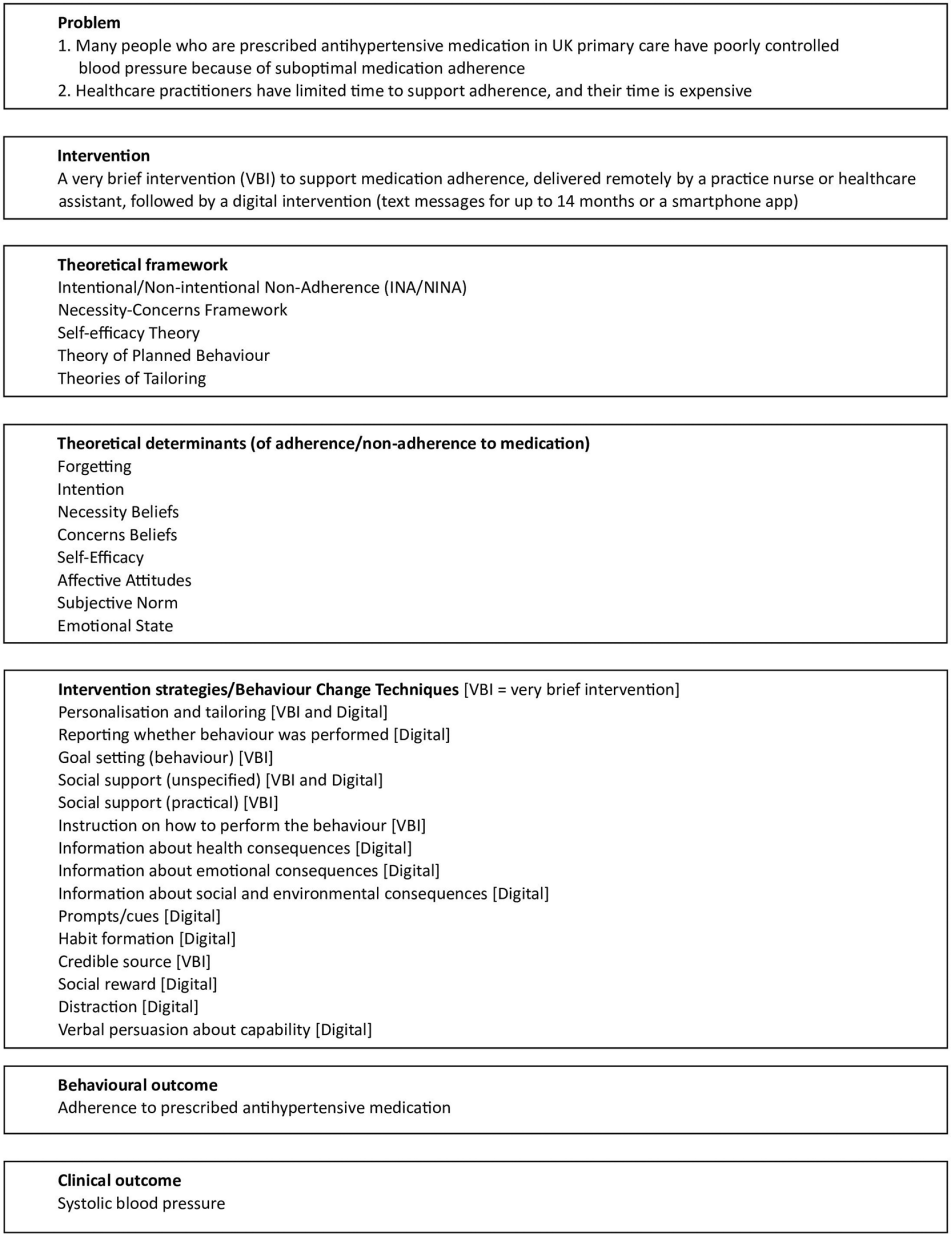
Logic model for the PAM intervention.

The PAM intervention is a theory-based intervention based on the distinction between intentional and non-intentional non-adherence (INA and NINA), ^28– 31^ the necessity-concerns framework, ^32^ self-efficacy theory, ^33^ the theory of planned behaviour ^34^ and theories of tailoring. ^35^ The intervention uses relevant behaviour change techniques (BCTs ^36^) and was informed by our experience of designing and evaluating brief face-to-face and digital interventions for behaviour change, including medication adherence ^37– 44^ and by extensive reviewing and development work. ^45– 48^ It comprises two components: a VBI delivered by the practitioner remotely using a telephone or video call, lasting no more than five minutes, followed by a digital intervention. (See Figure 2 for the logic model.)

In the VBI, the practitioner emphasises to the participant that it is very important that they take all their medications as prescribed. The practitioner then introduces the digital interventions, telling the participant that this would involve free support to take their medications in the form of a text messaging programme or a smartphone app (Android only). To receive the messages or notifications (which will be personalised and tailored), they will need to answer a few more questions (e.g. would they prefer to receive text messages or use the app, what is their mobile phone number, which medication would they like to be reminded about).

Starting the day after the baseline consultation (for text messaging) or the day of downloading and installing the app (for app users), participants will receive automated messages or notifications for up to 420 days, at least one a day on most days, including query messages or notifications asking them to provide a response. Based on information participants provide digitally at baseline and during the intervention, they will receive individually-tailored messages designed to address INA and/or NINA. INA is addressed with messages to reinforce necessity beliefs about medications (e.g. “Even if you do not feel any different after taking each of your pills, you can keep your blood pressure under control when you take your meds regularly”), and to counter concern beliefs (e.g. “Ask your GP about common side effects associated with your meds. If you are experiencing any of these, you can ask for advice on how to cope with them”). NINA is addressed through reminder messages (e.g. “Don’t forget to take your medication today: Ramipril, 2 tablets, 1.25 mg, at 16:00”), though INA messages may also serve as implicit reminders. The content of the reminders will be updated if changes are made to the participant’s prescribed medication during the intervention period. In the baseline consultation, the participant will be asked to specify their preferred times for receiving the reminders and the other messages. Query messages/notifications asking whether the participant has taken all their prescribed medications in the last seven days will be sent weekly. Other behaviour change techniques ^36^ included in the intervention are verbal persuasion about capability, information about health/emotional/social and environmental consequences, social reward, habit formation and distraction.

Although the app and text messaging interventions were designed to provide broadly comparable information and support, the app includes several features that are not available in the text messaging. For example, participants can view a bar chart of their adherence over the past one week or one month based on their responses to the query notifications. They can also enable or disable medication reminders.

Participants will be given several opportunities to stop the messages/notifications by sending a STOP message or notification to the system. Intervention participants who opt to receive text messages will be given several opportunities to switch to the app if they wish; however, app users will not be invited to switch (or switch back) to receiving text messages. The control group will receive usual care only i.e. no VBI, no digital intervention.

### Measures

Systolic blood pressure

The primary outcome of systolic BP will be based on BP readings self-measured by participants at home using validated automated electronic sphygmomanometers (A&D Medical UA-611) with 30 memories function ^49^ provided by the research team. After their baseline consultation and again at 12 months, participants will be posted a BP monitor, an information leaflet, a paper template on which to record their BP measurements and a link to a video demonstrating how to perform the BP measurement. They will be contacted by a member of the research team, blinded to group allocation, to facilitate return of the remote measurements. At each timepoint, they will be asked to take eight BP measurements over two days (two measurements on two mornings and two evenings with a two minute gap between each measurement), record their BP readings on the template, and post the BP monitors with the template to the research team. As an alternative to posting the template, they will be able to report their readings online using a secure web portal.

Data from the BP monitors (linked to the participant using a unique code) will be extracted by a member of the research team blinded to group allocation.

In addition, BP measurements will be extracted from practice records (up to two readings in the 12 months before randomisation and one reading that is closest to 12 months post-randomisation and no more than nine months prior to this timepoint).

Medication adherence

Medication adherence will be measured by chemical adherence testing (CAT) of urine samples and by self-report.

CAT detects the presence or absence of antihypertensive medication (or their metabolites) in urine. The period of detection is about 4-6 half lives of a medication. Participants will be posted a urine sample kit, a template and a printed information sheet after the baseline consultation and again at 12 months. They will be asked to provide spot urine samples (10 ml) early in the morning, record the name and the dosage (in mg) of all their BP lowering medications on the template, and post securely using special delivery the sample and template to the University Hospitals of Leicester National Centre for Drug Adherence Testing for analysis on the same morning.

Urinalysis will be conducted by liquid chromatography-tandem mass spectrometry (LC-MS/MS), which is highly sensitive and specific. Adherence will be defined in terms of the presence or absence of antihypertensive medications (or their metabolites) in the urine sample and, more specifically, by comparing the medications detected in the urinalysis with those reported by the participant. For the latter, total adherence to antihypertensive medication is defined if all daily reported medications (or their metabolites) are detected in the urinalysis, partial adherence if fewer medications than reported are detected, and total non-adherence if no reported medications are detected. ^26, 50^

Self-reported medication adherence will be assessed by the Medication Adherence Report Scale (MARS). ^51^ Two MARS items will be used at baseline in the baseline consultation. The first item assesses non-intentional non-adherence: “I forget to take my tablets”; the second assesses intentional non-adherence: “I alter the dose of my tablets”; for both items, the response options are: Always, Often, Sometimes, Rarely, Never. In addition, after the baseline consultation and again at 12 months, participants will be asked to complete the full five-item MARS scale which includes these two items. At baseline (during the baseline consultation) and 12-month follow up, participants will be asked two additional questions: “How much of your prescribed tablets have you taken in the last month?”, with responses from 0% to 100% ^52^; and “How many days in the past week have you taken all your prescribed tablets”, with responses from 0 to 7 days. ^44^

Practice level measures

Geographical region and Index of Multiple Deprivation derived from the practice postcode ^27^ will be recorded.

Additional clinical outcomes

Diastolic BP will be measured after the baseline consultation and at 12 months using the same methods as for systolic BP above. Haemoglobin A1c and full lipid profiles (triglycerides, cholesterol, HDL cholesterol, LDL cholesterol) will be measured after the baseline consultation and at 12 months for patients who report having type 2 diabetes and/or high cholesterol in addition to hypertension. Participants will be sent finger prick test kits and asked to collect home blood samples for one or both clinical outcomes and post the samples to The Doctors Laboratory ( www.tdlpathology.com) for analysis.

In addition, blood sample test results collected in the practice prior to baseline and close to the scheduled 12-month follow up timepoint for haemoglobin A1c and full lipid profile will be obtained from practice records.

Additional measures

Sociodemographic information will be collected from participants at baseline (sex; age and date of birth; ethnicity; postcode for Index of Multiple Deprivation ^27^). Information about medications the participant is taking will be collected during the baseline consultation, from practice records and on the post-baseline and 12-month follow-up questionnaires.

The time to complete each section of the baseline consultation will be recorded automatically by the online system. For intervention participants, measures collected during the intervention via text messaging or the app will include confidence in taking all their prescribed medications, beliefs about the consequences of medication adherence and whether they have taken all their prescribed medications in the last seven days. These will be used for tailoring the messages/notifications. The Beliefs About Medicines Questionnaire (BMQ) ^53^ will be completed after the baseline consultation and at 12-month follow up. Health behaviours related to hypertension will be measured at 12 months via five single items measuring salt consumption, alcohol consumption, diet, physical activity and smoking. ^54– 58^ For intervention participants, the 12-month questionnaire will also include questions about the use, acceptability, usefulness and perceived effectiveness of the two intervention components.

Process evaluation

To assess the fidelity of delivering the very brief intervention, each practitioner will be asked to randomly select at least three of their consultations and audio record them. The full set of audio recordings will include intervention and control consultations. Two independent coders will use a checklist to code the transcripts for adherence to the recommended intervention delivery.

To explore implementation of, and engagement with, the intervention and its different components, semi-structured interviews will be conducted with practitioners and participants by telephone: 1) A purposive sample of 10-15 practitioners will be interviewed after they have conducted all their baseline consultations; the interviews will assess their views on the research procedures and intervention implementation; 2) A sample of 35-40 participants, including those in the intervention group and the control group, will be interviewed after the 12-month follow up to explore their views of the intervention and other aspects of the research procedures. Analysis of the transcribed interviews will follow Braun and Clarke’s six-step thematic approach ^59, 60^ aided by NVivo software. ^61^

Log files recording use of the digital interventions will also be analysed. Measures will include the number of messages/notifications sent and received, the proportion of query messages/notifications to which participants responded, and whether and when they stopped the intervention.

### Sample size

A sample size for analysis of 434 patients (217 per arm) who have complete data on systolic BP at 12 months will give 90% power to detect a 5 mmHg difference in mean systolic BP between arms, assuming a standard deviation of 16 mmHg (a conservative estimate based on the feasibility trial ^22^) and using a two-sided t-test at the 5% significance level. A 5 mmHg difference in systolic BP is of clinical significance, being associated with a 20% reduction in major vascular events. ^62– 64^ Assuming that 20% of participants do not provide systolic BP data at 12 months, 542 patients (271 per arm) will need to be randomised.

### Statistical analysis

In the analyses, an intention to treat (ITT) approach will be used, maintaining participants in the arm to which they were randomised.

For the primary outcome analysis, a linear mixed effects model will be used to compare the mean systolic BP at 12 months between the two arms and quantify this as a difference in means and 95% confidence interval. A p-value for this powered outcome will be reported and assessed against the two-sided 5% significance level. The outcome for this analysis will be 12-month systolic BP self-measured by monitor at home. But if there are missing data on this measure, an additional source of BP measures will be used, namely systolic BP measures from practice records closest to 12 months post-randomisation.

In a supportive model, there will be baseline covariate adjustment for sex, age, geographical region, the closest measure prior to randomisation of systolic BP obtained from practice records, and for the randomisation stratifier of practitioner as a random effect. Missing data in covariates will be handled using the missing indicator method. ^65^

A sensitivity analysis will be undertaken to examine the robustness of the main model analysis result to the ‘missing at random’ (MAR) assumption. ^66^ The aim is to adequately explore the impact of departures from the MAR assumption on the primary outcome results. This will be handled first within a ‘not-missing-at-random’ sensitivity analysis considering optimistic and pessimistic scenarios for the intervention effect size by arm in those with missing data, with results displayed graphically, as in a previously analysed trial. ^67^ Second, the impact will be explored by incorporating baseline predictors of primary outcome missing status that are differential by arm.

We will also conduct a Complier Average Causal Effect (CACE) analysis by defining a ‘compliant’ per protocol population analysis in those participants who did not stop the digital intervention in the first three months and corresponding ‘would-be compliers’ in the control arm. ^68, 69^

Secondary outcomes (including the other clinical outcomes and measures of medication adherence), being continuous, will be analysed using mixed models and interpreted from 95% confidence intervals.

Pre-specified subgroup variables will be examined in relation to the primary outcome and will involve an initial, indicative, test of differential intervention effect across the subgroup variable before summarising the intervention effect in each of two subgroup categories. The proposed subgroup variables comprise: sex; age group; geographical region; and deprivation (high versus low, based on the Index of Multiple Deprivation, derived from the patient’s home postcode ^27^). Assuming a sample size for analysis of 434 and equal sized categories, the intervention effect observed in each subgroup will be estimated with a 95% confidence interval having an informative width of +/- 4.3 mmHg.

### Economic analysis

Overview

We will undertake a detailed analysis of the cost and cost-effectiveness of the PAM intervention compared with usual care alone using accepted economic evaluation methods. ^70^ We will estimate cost and cost-effectiveness for the within-trial period (12 months) and also over the expected lifetime of the patient. Costs will be assessed from the perspective of the NHS and personal social services (PSS). Cost components included in the analysis will consist of the detailed cost of the VBI and digital interventions, antihypertensive and other medications, use of NHS and social services, and costs incurred by patients. Unit costs will be taken from standard published sources.

Within-trial analysis

The cost-effectiveness measures in the within-trial analysis will be the incremental cost per change in systolic BP and the incremental cost per quality-adjusted life year (QALY) gained. Costs will be measured as described. During the within-trial period, resource use will be collected via retrospective questionnaire at six and 12 months covering the preceding six-month period. QALYs will be calculated based on the health related quality of life (HRQL) and mortality data collected during the trial. HRQL will be measured using the EQ-5D-5L ^71^ ( euroqol.org), collected at post-baseline, six months and 12 months for each patient. Utility scores will be calculated from the EQ-5D-5L descriptive system profiles using the recommended algorithm for the UK population at the time of analysis.

QALYs will be calculated for individual participants using an area-under-the curve approach, with linear interpolation between time points (post-baseline, 6 months, 12 months). Missing values of EQ-5D-5L and resource use will be imputed using multiple imputation incorporating variance correction factors.

The incremental cost-effectiveness ratio (ICER) will be calculated as the mean incremental cost divided by the mean QALYs gained for the intervention arm compared with usual care. Bootstrapped estimates of the mean differences in cost and QALYs will be used. ^72^ The incremental net monetary benefit (INMB) will be calculated as the difference in mean QALYs between the intervention and usual care arms, multiplied by the threshold value, minus the corresponding difference in mean costs. A cost-effectiveness acceptability curve (CEAC) will be constructed to show the probability that the PAM intervention is cost-effective at 12 months compared with usual care across a range of willingness-to-pay (WTP) thresholds. Probabilistic sensitivity analysis will be used to assess the impact of key assumptions on cost-effectiveness.

Long-run analysis

In the long-run analysis, cost-effectiveness will be calculated in terms of the incremental cost per QALY gained for the PAM intervention versus usual care. The analysis will incorporate and then extrapolate from the 12-month time period of the within-trial analysis. We will use a Markov model to estimate the long-term cost-effectiveness of the PAM intervention which will be based on a previous model from the TASMINH2 study. ^73^ This will account for age-related risks of CVD events over the life course as a function of systolic BP levels, which will in turn be influenced by adherence to antihypertensive medications. Costs and utility scores obtained from published studies will be assigned to health states. The analysis will assume varying durations of effect of the PAM intervention beyond those observed at 12 months on medication adherence and therefore systolic BP. We will undertake deterministic (one-, two- and multi-way) and probabilistic sensitivity analysis (including constructing a cost-effectiveness acceptability curve) assuming appropriate distributions and parameter values. ^74^

### Trial dates

Participants were recruited between January 2021 and March 2022.

## Ethics and dissemination

The study was approved by the East of England Cambridge East Independent Research Ethics Committee (REC reference 19/EE/0354, 05/02/2020), the Health Research Authority (HRA) and Health and Care Research Wales (HCRW).

The findings will be disseminated to the scientific community, participating practitioners and the public using a range of methods, including journal articles, conference presentations and the programme website. Primary care practices will disseminate aggregate results to the trial participants.

## Data Availability

No underlying data are associated with this article. Open Science Framework: SPIRIT Checklist 20.10.25. https://doi.org/10.17605/OSF.IO/AJBNX ^75^ This project contains the following underlying data:
• SPIRIT Checklist • Patient information sheet • Patient consent form Data are available under the terms of the Creative Commons Attribution 4.0 International license (CC-BY 4.0).
