# Supplementary material for: Randomised controlled trial of a very brief nurse-delivered intervention followed by a digital intervention to support medication adherence and reduce blood pressure in people prescribed treatment for hypertension in primary care: protocol for the Programme on Adherence to Medication (PAM) trial
Source: NIHR Open Res. 2026 Feb 25;6:18. [Version 1] doi: 10.3310/nihropenres.14136.1 (PMC13090759; doi:10.3310/nihropenres.14136.1)
Supplement: Peer review history

# Reviewer report: Reviewer response for version 1

**DOI:** 10.3310/nihropenres.15381.r40138

**Published:** 2026-04-17

**Authors:** Russell Jude de Souza

**Affiliations:** 1 McMaster University, Hamilton, Ontario, Canada

The authors will conduct a two-arm, multicentre, individually randomised controlled parallel-group superiority trial in primary care practices across England and Wales. The study will recruit 542 patients who are prescribed antihypertensive medication and have poorly controlled blood pressure. Participants will be randomly allocated to either an intervention group or a usual care control group. Participants in the intervention group will receive a very brief intervention delivered remotely by a practice nurse or healthcare assistant via telephone or video call, followed by a digital intervention using text messaging or a smartphone app, in addition to usual care. Control group participants will receive usual care alone. The primary outcome will be systolic blood pressure measured at 12 months. Medication adherence will be assessed using both chemical adherence testing of urine samples and self-reported measures. The trial will also include an economic evaluation and a process evaluation to assess implementation and mechanisms of impact.

 I have a few suggestions, and some comments

 1. What is the value of both the verbal and written consent? This seems like it may be difficult to track-- and response rates may be low. What would the value be of someone providing verbal and written consent? Or is the written option only for those who refuse to provide verbal consent? Please clarify.  

 2. Some details on who will be developing and delivering the online content for the training session would be helpful.

 3. The two-way communication is an excellent approach.

 4. The sample size and statistical analysis plan are appropriately presented.

Is the study design appropriate for the research question?

Yes

Is the rationale for, and objectives of, the study clearly described?

Yes

Are sufficient details of the methods provided to allow replication by others?

Yes

Are the datasets clearly presented in a useable and accessible format?

Not applicable

Reviewer Expertise:

diet, randomized trials, evidence synthesis, nutrition epidemiology

I confirm that I have read this submission and believe that I have an appropriate level of expertise to confirm that it is of an acceptable scientific standard.

# Reviewer report: Reviewer response for version 1

**DOI:** 10.3310/nihropenres.15381.r40141

**Published:** 2026-04-03

**Authors:** Sonia Blanco Mejia

**Affiliations:** 1 Nutritional Sciences, University of Toronto,, Toronto,, ON M5S 1A8,, Canada

The PAM protocol was well written and contained all the applicable SPIRIT checklist items. The study was well planned and provides sufficient information for its replication. A suggestion is to add the stage of data collection, as it seems from the recruitment dates that all data should have been collected by now. There are a few things that might need further explanation. 

 1.- Would you be collecting or did you collect data on with whom these individuals live (alone, with a partner, with a family member, in an assisted home, etc)? This information might be useful on medication adherence or effectiveness of the app/text messages.

 2. - Participants and recruitment. Page 6. For exclusion, point c states that if they have had a recent severe life-threatening event OR are under treatment for a long-term health condition apart from hypertension, high cholesterol or type 2 diabetes. Could you elaborate more on this? Provide at least a few examples of what you considered exclusion of a long term health condition.

 3.- Participants and recruitment. Page 6. For point e, would this be specific to your country? whom else could manage their BP other than their GP?

 4.- Intervention, page 8.  Were participants reminded about ALL their current medications or only BP medications? 

Is the study design appropriate for the research question?

Yes

Is the rationale for, and objectives of, the study clearly described?

Yes

Are sufficient details of the methods provided to allow replication by others?

Yes

Are the datasets clearly presented in a useable and accessible format?

Not applicable

Reviewer Expertise:

RCTs, SRMAs, Nutrition, CVD risks, obesity, HTN, diabetes

I confirm that I have read this submission and believe that I have an appropriate level of expertise to confirm that it is of an acceptable scientific standard.
